# Biotic interactions between benthic infauna and aerobic methanotrophs mediate methane fluxes from coastal sediments

**DOI:** 10.1093/ismejo/wrae013

**Published:** 2024-01-31

**Authors:** Elias Broman, Markus Olsson, Adele Maciute, Daniel Donald, Christoph Humborg, Alf Norkko, Tom Jilbert, Stefano Bonaglia, Francisco J A Nascimento

**Affiliations:** Department of Ecology, Environment and Plant Sciences, Stockholm University, Stockholm 10691, Sweden; Baltic Sea Centre, Stockholm University, Stockholm 10691, Sweden; Department of Ecology, Environment and Plant Sciences, Stockholm University, Stockholm 10691, Sweden; Department of Marine Sciences, University of Gothenburg, Gothenburg 41390, Sweden; Tvärminne Zoological Station, Faculty of Biological of Environmental Sciences, University of Helsinki, Helsinki 10900, Finland; Baltic Sea Centre, Stockholm University, Stockholm 10691, Sweden; Tvärminne Zoological Station, Faculty of Biological of Environmental Sciences, University of Helsinki, Helsinki 10900, Finland; Baltic Sea Centre, Stockholm University, Stockholm 10691, Sweden; Tvärminne Zoological Station, Faculty of Biological of Environmental Sciences, University of Helsinki, Helsinki 10900, Finland; Tvärminne Zoological Station, Faculty of Biological of Environmental Sciences, University of Helsinki, Helsinki 10900, Finland; Environmental Geochemistry Group, Department of Geosciences and Geography, Faculty of Science, University of Helsinki, Helsinki 00014, Finland; Department of Marine Sciences, University of Gothenburg, Gothenburg 41390, Sweden; Department of Ecology, Environment and Plant Sciences, Stockholm University, Stockholm 10691, Sweden; Baltic Sea Centre, Stockholm University, Stockholm 10691, Sweden

**Keywords:** animals, coastal, RNA, methane oxidation, climate change, bioturbation

## Abstract

Coastal ecosystems dominate oceanic methane (CH_4_) emissions. However, there is limited knowledge about how biotic interactions between infauna and aerobic methanotrophs (i.e. CH_4_ oxidizing bacteria) drive the spatial–temporal dynamics of these emissions. Here, we investigated the role of meio- and macrofauna in mediating CH_4_ sediment–water fluxes and aerobic methanotrophic activity that can oxidize significant portions of CH_4_. We show that macrofauna increases CH_4_ fluxes by enhancing vertical solute transport through bioturbation, but this effect is somewhat offset by high meiofauna abundance. The increase in CH_4_ flux reduces CH_4_ pore-water availability, resulting in lower abundance and activity of aerobic methanotrophs, an effect that counterbalances the potential stimulation of these bacteria by higher oxygen flux to the sediment via bioturbation. These findings indicate that a larger than previously thought portion of CH_4_ emissions from coastal ecosystems is due to faunal activity and multiple complex interactions with methanotrophs.

## Introduction

Methane (CH_4_) concentrations in the atmosphere have increased sharply in recent decades (e.g. from 1645 ppb to 1908 ppb during years 1984–2022) [1–3]. Emissions of CH_4_ from microbial metabolic processes in the Earth surface system have been estimated to be a major contributor to rising atmospheric CH_4_ concentrations [4]. In reducing environments within aquatic ecosystems, methanogenesis is widespread, and aquatic systems globally contribute up to half of all global CH_4_ emissions [5]. Marine shallow waters, such as coastal environments, are estimated to account for 0.8–3.8 Tg CH_4_ yr^−1^ of the oceanic CH_4_ emissions (6–12 Tg yr^−1^) even though coastal areas only constitute <3% of the oceans [6]. In the water and sediment, CH_4_ can be removed through oxidation by CH_4_ oxidizing microorganisms (so-called methanotrophs). Aerobic methanotrophs reside in the oxic water and sediment surface, and have been shown to potentially oxidize up to half of all CH_4_ in coastal waters [7], while anaerobic CH_4_ oxidizing archaea (ANME) have been shown to oxidize up to 90% of CH_4_ produced in deep anoxic sediment [8, 9]. Because aerobic methanotrophs inhabit the sediment surface where oxygen is available [10], they are exposed to both small and large animals that not only graze on bacteria but also influence their environment by reworking sediment particles through bioturbation, here used to also include bioirrigation [11–14]. However, it is unknown how benthic organisms influence CH_4_ oxidation rates or the abundance and metabolic activity of methanotrophs. This is important to understand, because climate change and other anthropogenic stressors directly affect coastal biodiversity [15] and this, in turn, could alter coastal CH_4_ fluxes significantly.

Although the role of bioturbation by macrofauna (invertebrates >1 mm body size) in modulating biogeochemical cycles has been well studied [12, 16], the role of meiofaunal bioturbation is not well understood. Meiofauna are microscopic invertebrates (0.04–1 mm body size), such as nematode worms, inhabiting the sediment surface, where oxygen concentrations are high enough to support the survival of most taxa [17]. Meiofauna have been shown to increase overall oxygen uptake by 3–33% in marine sediments [18], as well as sediment porosity and transport of solutes [19, 20]. Meiofauna feed on several sources of sedimentary organic matter (OM), including bacteria and algae [14], and, in turn, can stimulate the overall rate of OM degradation [21]. Higher abundance of meiofauna has therefore been shown to alter bacterial community structure [21, 22] and increase bacterial denitrification [23]. However, there is a knowledge gap on whether the abundance of meiofauna can influence CH_4_ sediment-to-water fluxes and the abundance and activity of methanotrophs that are also found on the sediment surface.

The larger macrofauna may alter CH_4_ cycling dynamics. For example, bivalves and polychaetes have been found to increase CH_4_ fluxes in coastal sediments [24], presumably through increased advection and presence of methanogenic symbionts. Macrofauna can also control meiofauna populations through predation [13] and competition for food [13, 25, 26], as well as by bioturbation and modulation of oxygen concentrations in the sediment [13]. These types of macrofauna–meiofauna–microbe interactions might indirectly alter methanotroph abundance due to decreased bacterial grazing by meiofauna. As bioturbation by macrofauna alters geochemical properties of the sediment, this process has also been shown to affect the prokaryotic community structure directly [27]. Macrofauna might thus influence methanotroph abundance and activity as these bacteria prefer oxic-anoxic interfaces in the sediment, where both O_2_ and CH_4_ are available [10]. Furthermore, some macrofauna, such as many bivalves, are filter and deposit feeders. Thus, it is likely that particle-attached bacteria are also consumed [28, 29]. It is still not understood, however, if and how the interactions of both meio- and macrofauna influence methanotrophs and CH_4_ oxidation rates.

The aim of this study was to investigate experimentally whether and how macrofauna and meiofauna influence CH_4_ fluxes, CH_4_ oxidation rates, and abundance and activity of aerobic methanotrophs in coastal sediments (i.e. macrofauna–meiofauna–bacteria interaction). For this purpose, we collected sediment cores and manipulated the abundance of meiofauna (low or high meiofauna abundance) and macrofauna (low or high meiofauna abundance, with or without macrofauna). We measured variables such as CH_4_ pore-water concentrations, oxidation rates, and sediment–water flux. We coupled these results with measurements of sediment oxygen penetration depth, meiofauna abundance, macrofauna biomass, and total RNA-seq that were used to investigate changes in the relative abundance of aerobic methanotrophs and their transcriptional activity of genes related to CH_4_ oxidation. We hypothesized that increased oxygen availability due to bioturbation by higher abundance of meiofauna, and the presence of macrofauna, would increase CH_4_ oxidation rates, and the relative abundance of methanotrophs and RNA transcripts related to CH_4_ oxidation.

We found that macrofauna in coastal sediments increase CH_4_ fluxes via bioturbation, while meiofauna slightly offset this flux. The CH_4_ flux decreased CH_4_ pore-water availability which resulted in lower abundance and activity of aerobic methanotrophs. These findings indicate that sediment containing macrofauna enhanced CH_4_ fluxes 2–6 times, with the highest fluxes in sediment with macrofauna and low meiofauna abundance. Our findings show that a substantial part of CH_4_ emissions from shallow marine waters is due to faunal activity and multiple complex pathways of interactions with methanotrophs.

## Results

### Validation of the experimental design: infauna abundance and biomass in the treatments

**Figure 1:**
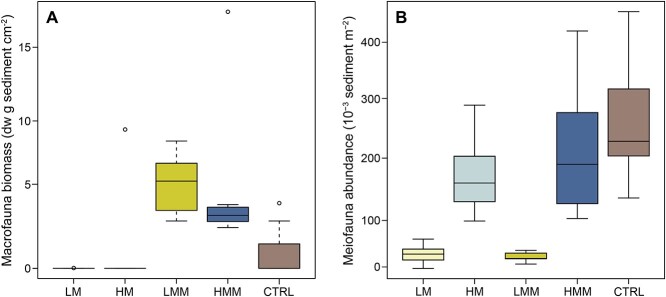
**Macrofauna biomass and meiofauna abundance across treatments.** Collected sediment cores were manipulated in terms of meiofauna and macrofauna (bivalve *M. balthica*) abundance and acclimated for 10 d. Treatments include: LM = low meiofauna, HM = high meiofauna, LMM = low meiofauna + macrofauna, HMM = high meiofauna + macrofauna, and unmanipulated sediment cores as a control (CTRL). (A) Biomass estimated from macrofauna collected from the sediment cores (0–14 cm sediment depth, *n* = 7 per treatment). (B) Meiofauna abundance estimated in the 0–1 cm sediment surface (*n* = 7 per treatment). The middle lines in each box denote the median, the bottom and top of the box show the first and third quartiles, and the whiskers show the maximum and minimum values. The circles denote outliers ≥1.5 × box length.

A total of 60 sediment cores were manipulated in terms of meiofauna and macrofauna abundance and distributed among five treatments. In brief, sediments were sequentially sieved, meiofauna were then isolated using a density extraction method [30] and added back at a higher concentration to a portion of the cores. To manipulate the macrofauna abundance, the bivalve *Macoma balthica* was added to half of the low and high meiofauna abundance cores. Treatments included: low meiofauna (LM), high meiofauna (HM), low meiofauna + macrofauna (LMM), high meiofauna + macrofauna (HMM), and unmanipulated sediment cores as a control (CTRL) (*n* = 12 independent sediment cores for each treatment). After 10 d of acclimation in aerated bottom water, the cores were used or sliced for various data collection (as detailed in the results below). There was significantly higher biomass of macrofauna in the LMM and HMM treatments compared with LM and HM (non-parametric Dunn tests with Benjamini–Hochberg p-adjustment, *P* < 0.05, *n* = 7 per treatment; Fig. 1A). Meiofauna abundance was significantly higher in the HM and HMM treatments compared with LM and LMM (on average 4 times higher, *n* = 7 per treatment, one-way ANOVA, *F* = 12.9, *P <* 0.001 with *post hoc* Tukey tests, *P <* 0.05); Fig. 1B). The meiofauna abundance and macrofauna biomass in the unmanipulated cores (i.e. CTRL) were within the range of our manipulated treatments and indicated that our experimental animal abundances were realistic to occur *in situ*.

### CH_4_ pore-water concentrations, CH_4 _oxidation rates, and methanotrophic activity decreased in meio- and macrofauna treatments

**Figure 2:**
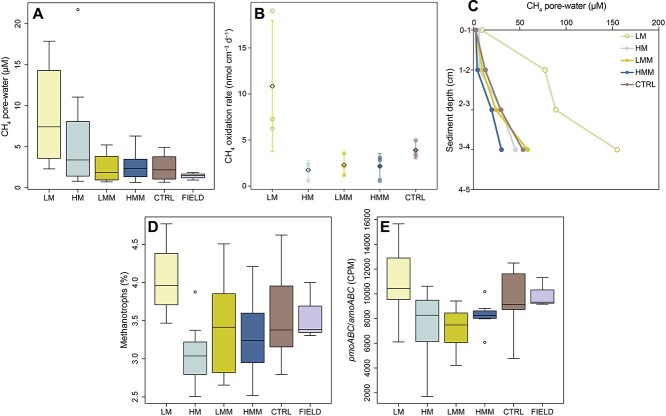
**CH**_**4**_ **pore-water concentrations, CH**_**4**_ **oxidation rates, CH**_**4**_ **pore-water profiles, relative abundance of aerobic methanotrophs, and RNA transcripts encoded by genes for CH**_**4**_ **oxidation.** (A) CH_4_ pore-water concentrations in the top 1 sediment surface (treatments *n* = 8, field *n* = 3). (B) CH_4_ oxidation rates based on injecting ^14^CH_4_ tracer in the top 0–2 cm sediment layer (*n* = 3 per treatment). The dots denote individual incubated sediment cores, diamonds denote mean values, and the error bars show SD. (C) CH_4_ pore-water profiles in the sediment. The 0–1 cm data are based on the average from all sliced cores (*n* = 8 per treatment), and the other depths were measured from one core per treatment (*n* = 1). (D) Total RNA-seq results from the top 1 cm sediment surface showing the 16S rRNA relative abundance (%) of methanotrophs (*n* = 8 per treatment). (E) The summed number of *pmoA*, *pmoB*, and *pmoC* transcripts in the top 1 cm sediment surface normalized as CPM reads (*n* = 8 per treatment). Note that ca 92% of the *pmoABC*/*amoABC* transcripts were estimated to be attributed to pmo (more details in Results).

Pore-water CH_4_ concentrations in the top 1 cm sediment were different among treatments (one-way ANOVA, F_(4,35)_ = 3.35, *P <* 0.05). Average CH_4_ in pore-water was highest in the LM treatment (8.8 ± 6.1 μM CH_4_), and was significantly higher than LMM (2.4 ± 17 μM CH_4_, *P =* 0.033) and CTRL (2.4 ± 1.6 μM CH_4_, ANOVA with Tukey tests, *P =* 0.042) (values show mean ± SD; Fig. 2A). Compared with LM, the high meiofauna treatments (HM and HMM) had on average lower CH_4_ pore-water concentrations but had more variation in the data and were not significantly different (HM 6.0 ± 7.1 μM and HMM 2.7 ± 1.8 μM CH_4_). CH_4_ pore-water concentrations in the field samples (μM 1.4 ± 0.5 CH_4_) showed less variation compared with the treatments but were not significantly different when compared with the treatments (ANOVA with Tukey tests, Fig. 2A). CH_4_ oxidation rates measured in the top 0–2 cm sediment were also impacted by treatment (one-way ANOVA, *F*_(4,10)_ = 3.92, *P* < 0.05). CH_4_ oxidation rates were highest in the LM treatment (10.8 ± 7.1 nmol oxidized CH_4_ cm^−3^ d^−1^) compared with the other treatments (which had on average < 4 nmol oxidized CH_4_ cm^−3^ d^−1^, *n* = 3 per treatment) (Fig. 2B), with LM being significantly higher than HM (one-way ANOVA with *post hoc* Tukey tests, *P <* 0.05). CH_4_ turnover (i.e. amount of ^14^CH_4_ converted to ^14^CO_2_) had higher values in the LMM and HMM treatment with an average of 49% and 67% CH_4_ turnover, respectively. However, CH_4_ turnover was not statistically significant compared with the other treatments (both LM and HM had on average 38%, and CTRL 66%) (Supplementary Fig. S1). LM had also the highest CH_4_ pore-water profile concentrations down to 4 cm sediment depth (155 μM CH_4_ at 4 cm), followed by LMM (58 μM), CTRL (53 μM), HM (45 μM), and HMM (30 μM) (one CH_4_ depth profile per treatment; Fig. 2C).

In accordance with higher measured CH_4_ oxidation rates, the LM treatment also showed the highest relative abundance of methanotrophs in the top 1 cm of sediment (4.0 ± 0.5%, based on extracting 16S rRNA reads from total RNA-seq data) compared with an average of <3.6% in the other treatments. Due to the high variation in the treatments amended with macrofauna, only HM (3.1 ± 0.4%) was statistically different compared with LM (ANOVA with Tukey tests, *P =* 0.007, *n* = 8 per treatment; Fig. 2D). The methanotrophs found in our data sets include *Methylomirabilota*, *Beijerinckiaceae*, *Methylococcales*, and *Methylacidiphilales* which were the known bacterial methanotrophic groups present in the samples. The relative abundance of methanotrophs in the CTRL and field samples were not different from the manipulated treatments (ANOVA with Tukey tests, Fig. 2D), indicating that the results from the treatments were within what could be expected *in situ*. Furthermore, the total RNA transcripts attributed to the genes *pmoA/amoA*, *pmoB/amoB*, *pmoC/amoC* in the KEGG database were significantly higher in LM (10 936 ± 2899 counts per million reads (CPM)) compared with HM and LMM (both on average < 7600 CPM, ANOVA with Tukey tests, *P <* 0.05, *n* = 8 per treatment; Fig. 2E). The number of *pmoABC/amoABC* RNA transcripts in the CTRL and field were not significantly different from the treatments, indicating that our transcripts numbers are within what could be expected *in situ* (ANOVA with Tukey tests) (Fig. 2E). The *pmoAB/amoAB* classified reads were annotated against the UniProtKB-SwissProt database to try and differentiate particulate methane monooxygenase (pmo) and ammonia methane monooxygenase (amo). The results showed that 92 ± 2% reads per treatment were attributed to pmo indicating that majority of the KEGG classified *pmoABC/amoABC* transcripts belonged to aerobic methanotrophs (Supplementary Fig. S2). Soluble methane monooxygenase (e.g. gene *mmoX*) had <5 CPM of RNA transcripts for each sample and was therefore not as an active metabolic process compared with pmo. Quantitative reverse transcription PCR (RT-qPCR) primers targeting 16S rRNA gave on average a threshold cycle (Ct) value of 13 for all treatments (LM 13.1, HM 12.8, LMM 13.0, HMM 12.8, CTRL 13.3), indicating that there was no large difference in bacterial 16S rRNA abundance. Finally, to investigate methanol conversion to CO_2_, oxic methane production, and methanogenic activity (or anaerobic methane oxidation via reverse-methanogenesis) in the sediment surface we, respectively, analyzed methanol dehydrogenase (*mxaF*), alpha-D-ribose 1-methylphosphonate 5-phosphate C-P lyase (*phnJ*), and methyl-coenzyme M reductase alpha subunit (*mcrA*) transcripts. The results showed that each treatment had on average 63–137 CPM *mxaF* transcripts with no statistical difference between treatments (one-way ANOVA with Tukey tests, *P* > 0.05). While *phnJ* (0–1 CPM) and *mcrA* (0–2 CPM) transcripts were close to zero in the treatments (Supplementary Fig. S3).

### CH_4_ flux and O_2_ penetration depth increased with the presence of meio- and macrofauna

**Figure 3:**
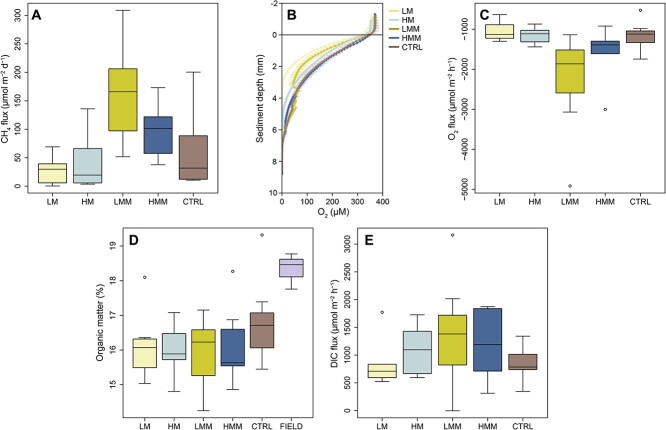
**CH**_**4**_ **sediment–water flux, O**_**2**_ **sediment penetration depth, O**_**2**_ **flux in the bottom water, OM content, and DIC sediment–water flux.** (A) CH_4_ sediment–water flux for each treatment (*n* = 7 per treatment, except HMM *n* = 6). (B) O_2_ concentration profiles in the sediment (*n* = 3 per treatment, error bars show SE). (C) O_2_ flux, i.e. consumption rate measured in the water phase on top the sediment surface (*n* = 7 per treatment, except HMM *n* = 6). (D) OM content (%) estimated in the top 1 cm sediment surface (treatments *n* = 7, field samples *n* = 3). (E) DIC sediment–water flux for each treatment (*n* = 7 per treatment, except LM and HMM *n* = 6).

There was a significant effect of treatment on CH_4_ sediment–water flux (one-way ANOVA, *F*_(4,29)_ = 5.39, *P <* 0.01). The LMM treatment was found to have the highest CH_4_ sediment–water flux (161.8 ± 91.9 μmol CH_4_ m^−2^ d^−1^) compared with the other treatments (which were on average < 100 μmol CH_4_ m^−2^ d^−1^), and this difference was statistically significant compared with treatments without macrofauna, LM and HM (ANOVA with Tukey tests, *P =* 0.003 and 0.013, respectively, *n* = 6–7 per treatment; Fig. 3A). HMM had on average slightly lower fluxes than LMM, whereas the meiofauna-only treatments (LM and HM) had on average lower fluxes than treatments amended with macrofauna (Fig. 3A). However, due to variation in cores including animals, these differences were not statistically significant. The measured CH_4_ fluxes for all treatments were within the range of the CTRL (ANOVA with Tukey tests, *P* > 0.05), indicating that the measured CH_4_ fluxes could be expected *in situ*.

The LM treatment had the lowest O_2_ penetration depth in the sediment (3.1 ± 0.4 mm) compared with the other treatments (Dunn tests, *n* = 3 per treatment; *P <* 0.005). This was followed by HM (6.0 ± 0.9 mm), LMM (7.1 ± 2.2 mm); CTRL (7.3 ± 1.6 mm), and HMM (7.7 ± 1.5 mm), of which LMM and HMM were significantly different from each other (Dunn tests, *P <* 0.01) (Fig. 3B). O_2_ flux (i.e. O_2_ consumption measured in the bottom water overlying the sediment) was the highest in the LMM treatment (−2305 ± 1310 μmol O_2_ m^−2^ h^−1^) and was statistically significant compared with all other treatments (on average < −1700 μmol O_2_ m^−2^ h^−1^ per treatment) (Dunn tests, *P <* 0.05, *n* = 6–7 per treatment; Fig. 3C). Measurements of OM % in the top 1 cm sediment surface did not show difference between treatments (~16% OM, one-way ANOVA with Tukey tests, *n* = 7 per treatment; Fig. 3D). Compared with field samples (*n* = 3) there was slightly less OM% in all treatments (ca 2% less) potentially due to the experimental setup (sediment slicing and sieving) and the enclosed nature of the sediment cores throughout the experiment (ANOVA with Tukey tests, *P <* 0.05; Fig. 3D). Treatments with added animals (HM, LMM, and HMM) showed higher dissolved inorganic carbon (DIC) sediment–water flux (on average > 1000 μmol DIC m^−2^ h^−1^) than LM and CTRL (on average < 900 μmol DIC m^−2^ h^−1^, *n* = 6–7 per treatment; Fig. 3E), but the high variation between replicates within the animal treatments did not yield a statistical significance.

### Treatments with infauna changed methanotrophic taxonomy and community structure

**Figure 4:**
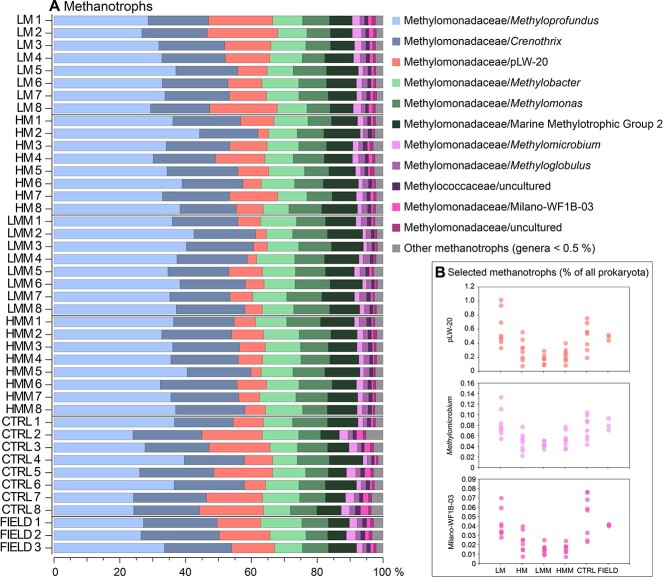
**Taxonomy of methanotrophic bacteria in the samples.** (A) Methanotrophic bacteria in the 0–1 cm sediment surface (extracted 16S rRNA from the total RNA-seq). Note that the x-axis shows the relative abundance (%) of the methanotroph community. (B) Abundant methanotrophic genera that were found to significantly decrease in treatments with higher abundance of meiofauna or added macrofauna.

In the top 1 cm sediment layer, the prokaryotic community was dominated by *Gammaproteobacteria* in all samples (~49% relative abundance, average of all samples), followed by *Bacteroidota* (~10%), and *Desulfobacterota* (~7%) (based on extracting 16S rRNA reads from the total RNA-seq data; Supplementary Fig. S4). The field samples showed a similar community composition of the main phyla compared with the treatments and CTRL, which indicated that the experimental design had not markedly altered the prokaryotic community (Supplementary Fig. S4). The methanotrophic community represented 2.5–4.8% of the prokaryotic community and was dominated by groups within *Methylococcales* (>90% relative abundance for each sample), and showed significant differences in genera between treatments (Fig. 4A). For example, the genera pLW-20, *Methylomicrobium,* and Milano-WF1B-03 had lower relative abundance in treatments with an elevated number of animals (HM, LMM, and HMM) when compared with LM (ca a 50% decrease, Dunn tests, *P <* 0.05; Fig. 4B). Together these genera represented ~14% of the methanotrophic community (average of all samples). Some of the most abundant methanotrophic genera did not change in relative abundance between treatments, such as *Methyloprofundus* and *Crenothrix* that together represented ~54% of the methanotrophic community (Dunn tests; Fig. 4A). The field samples showed a similar methanotrophic taxonomy to the treatments, indicating that our laboratory incubations had not changed specific groups of methanotrophs when compared with the field (Fig. 4A). To further confirm that there was a difference in methanotrophic genera between the treatments a non-metric multidimensional scaling (NMDS) multivariate analysis (based on the Bray–Curtis index) was conducted and showed that LM was different from HM and LMM (pairwise PERMANOVA tests, 9999 permutations, *P <* 0.05; Supplementary Fig. S5). That this difference in community structure was driven by methanotrophs that decreased in the animal treatments was confirmed with SIMPER analysis. On average there was an ~15% dissimilarity in Bray–Curtis beta diversity between LM and HM, LMM, HMM with the genus pLW-20 being the top methanotroph contributing to this dissimilarity (~5%, SIMPER analysis, *P <* 0.05).

### Structural equation modeling showed that mainly macrofauna enhances CH_4_ fluxes leading to lower CH_4_ pore-water concentrations and methanotrophic activity

**Figure 5:**
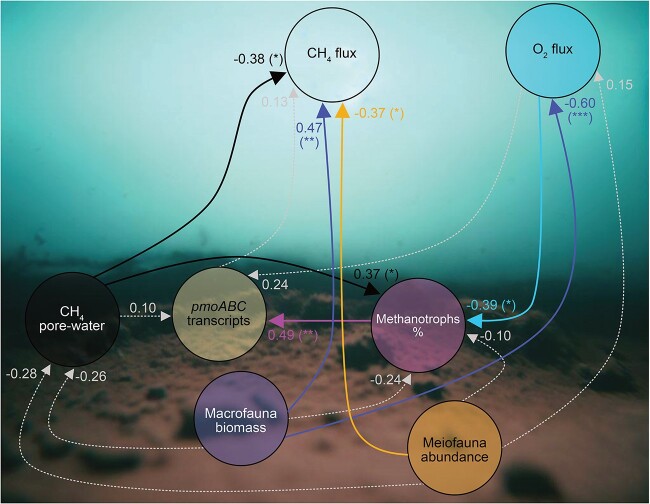
**SEM of investigated paths.** The model is based on data from all treatments collected in the study. Filled arrows show significant effects for each variable, and dashed arrows show non-significant effects. The numbers show standardized coefficients and the stars denote *P* values <0.05 (*), <0.01 (**), and <0.001 (***). Background image created via AI using Image Creator from Microsoft Designer powered by DALL-E 2.

A structural equation modeling (SEM) model including the analyzed data supported the proposed hierarchical structure (Fisher’s C = 6.4 and *P =* 0.78; Fig. 5 and Table 1). This was also the case for the most parsimonious model that identified similar significant paths (Fisher’s C = 22.2 and *P =* 0.44; Supplementary Fig. S6). The models showed that there was a direct effect of macrofauna biomass on CH_4_ fluxes (i.e. more macrofauna increases CH_4_ flux; standard coefficient = 0.47, *P <* 0.01), whereas there was no such relationship with macrofauna biomass on CH_4_ pore-water concentrations (standard coefficient = −0.26, *P* > 0.05). These findings were also shown when tested with spearman correlations (Supplementary Fig. S7). Meiofauna abundance had a significant negative relationship with CH_4_ flux (standard coefficient = −0.37, *P <* 0.05), driven by the high CH_4_ flux in the LM abundance treatments, particularly in LMM. However, macrofauna biomass and meiofauna abundance did not have a direct effect on the relative abundance of aerobic methanotrophs (standard coefficients = −0.24 and −0.10 with both *P* > 0.05, respectively), but macrofauna was indirectly related to this variable through its strong influence on O_2_ fluxes to the sediment (standard coefficient = −0.60, *P <* 0.001) and high sediment-to-water CH_4_ fluxes. CH_4_ pore-water concentrations and O_2_ fluxes were a strong significant driver of the abundance of methanotrophs (standard coefficients = 0.37 and −0.39 with both *P <* 0.05, respectively). The abundance of methanotrophs was, in turn, positively related to the number of *pmoABC* transcripts (standard coefficient = 0.49, *P <* 0.01).

**Table 1:** Detailed results from the SEM model.

**Predictor**	**Response**	***df***	**Std.Estimate**	*P* **value**	**Sig.**
CH_4_ pore-water (μM)	CH_4_ flux (μmol m^−2^ d^−1^)	30	−0.38	0.019	*
Macrofauna biomass (dw g sediment cm^−2^)		30	0.47	0.002	**
Meiofauna abundance (10^−3^ sediment m^−2^)		30	−0.37	0.017	*
*pmoABC* (CPM)		30	0.13	0.420	
Macrofauna biomass (dw g sediment cm^−2^)	CH_4_ pore-water (μM)	32	−0.26	0.124	
Meiofauna abundance (10^−3^ sediment m^−2^)		32	−0.28	0.101	
CH_4_ pore-water (μM)	*pmoABC* (CPM)	36	0.10	0.507	
Methanotrophs (%)		36	0.49	0.002	**
O_2_ flux (μmol m^−2^ h^−1^)		36	0.24	0.109	
Macrofauna biomass (dw g sediment cm^−2^)	O_2_ flux (μmol m^−2^ h^−1^)	32	−0.60	0.000	***
Meiofauna abundance (10^−3^ sediment m^−2^)		32	0.15	0.308	
Macrofauna biomass (dw g sediment cm^−2^)	Methanotrophs (%)	30	−0.24	0.206	
Meiofauna abundance (10^−3^ sediment m^−2^)		30	−0.10	0.566	
CH_4_ pore-water (μM)		30	0.37	0.031	*
O_2_ flux (μmol m^−2^ h^−1^)		30	−0.39	0.046	*

The standardized estimate (Std.Estimate) denotes the direction and strength of the relationship by the predictor variable on the response variable. The column sig. denotes the significance of the pathway with stars denoting *P* values <0.05 (*), <0.01 (**), and <0.001 (***). Note that negative O_2_ flux values indicate deeper oxygen penetration in the sediment.

## Discussion

### Macrofauna in the sediment accelerate CH_4_ transport

Our results provide evidence on how three-way interactions between macrofauna, meiofauna, and bacteria affect CH_4_ dynamics in marine sediments, elucidating the mechanisms through which benthic infauna enhances CH_4_ sediment–water fluxes in coastal ecosystems. The SEM and individually tested results show that macrofauna accelerate CH_4_ transport to the water column through bioturbation, thus reducing the concentration of pore-water methane in sediments. We found that sediment with added macrofauna increased the sediment–water CH_4_ flux on average 2–6 times depending on meiofauna abundance (with higher fluxes in sediment with low meiofauna abundance, comparing HMM and LMM with HM and LM, respectively). Our measured CH_4_ fluxes (0.3–309 μmol m^−2^ d^−1^, with the highest values in the macrofauna treatments) are within the range for other sites in the Baltic Sea, such as the southern Bornholm basin [31], southeastern Gdansk Deep [32], Vistula and Curonian lagoons [33], and the western Gotland Basin’s coast [34]. Compared with these studies from other locations in the Baltic Sea, our values are lower when compared with the high primary production season and/or hypoxic conditions at which CH_4_ fluxes can reach >1000 μmol m^−2^ d^−1^, and it is therefore possible that our measured macrofauna driven CH_4_ fluxes would have been even higher if the experiment had been conducted in late July or August instead of May. Compared with data from other coastal ecosystems, our measured sediment–water CH_4_ flux values are also in the range to what has been reported for habitats such as mangroves (279 μmol m^−2^ d^−1^), salt marshes (224 μmol m^−2^ d^−1^), and seagrass meadows (65 μmol m^−2^ d^−1^) [35]. However, none of these studies investigated the role of benthic infauna in mediating such fluxes. Our data show clearly that macrofauna biomass has an important effect on CH_4_ fluxes, significantly increasing these fluxes. The high CH_4_ fluxes in sediment with macrofauna lowered the availability of CH_4_ for aerobic methanotrophs in the sediment, resulting in a decrease in their abundance, activity, and corresponding CH_4 _oxidation rates. Although macrofauna also increased the oxygen supply to the sediment, potentially influencing methanotrophic activity, the SEM indicated that this effect was balanced by limited CH_4_ availability or too high O_2_ concentrations as at least some aerobic methanotrophs in coastal water have been shown to have a submicromolar O_2_ optimum [36]. In our experiment, the macrofauna treatments included an abundance of 1200 individuals m^−2^ sediment, which is in the range previously reported for the Baltic Sea [37, 38]. The introduced bivalve *M. balthica* is one of the most common animals in the Baltic Sea that dominates benthic infauna biomass in this system, and is known to be important in ecosystem functioning by e.g. enhancing benthic-pelagic coupling, increasing benthic respiration, and distributing fresh OM into the sediment via bioturbation [39]. Taken together, our results regarding macrofauna driven CH_4_ fluxes are therefore likely relevant for other coastal systems worldwide.

In addition to experiments that include live macrofauna [24, 40], experimentally induced mechanical bioturbation has been shown to increase CH_4_ fluxes [41, 42]. Such findings further support the idea that bioturbation in coastal sediments enhances CH_4_ fluxes. A previous experiment estimated CH_4_ emissions caused by macrofauna and showed that it represented 9.5% of the total CH_4_ emissions in the Baltic Sea [24]. In our study, macrofaunal enhanced CH_4_ fluxes were on average ~5 times higher compared with the previously reported values [24], likely due to our sediment being sampled further offshore and having higher *in situ* CH_4_ pore-water concentrations. As more macrofauna biomass has been shown to increase bioturbation rates [43], it is likely that other macrofauna than *M. balthica* also influences CH_4_ fluxes. For example, Bonaglia et al. [24] found that the polychaeta *Marenzelleria* also contribute to the CH_4_ sediment–water flux. Going further than these previous studies, here we also used measured CH_4_ oxidation rates and applied molecular tools to investigate the taxonomy and activity of aerobic methanotrophs and provide a mechanistic explanation. We found that the relative abundance of specific groups of methanotrophs, such as pLW-20, *Methylomicrobium,* and Milano-WF1B-03, were lower in treatments with macrofauna (i.e. LMM and HMM) presumably due to the lower concentrations of pore-water CH_4_. We did not detect a difference in methanol dehydrogenase activity (*mxaF* transcripts) between treatments, with a possible explanation being that methanotrophs and methylotrophs (i.e. methanol utilizing bacteria) use a similar methanol dehydrogenize enzyme [44]. Our results based on *pmoABC* transcripts and 16S rRNA therefore likely better reflect the methanotrophic community and activity in the sediment. It is also possible that this decrease in relative abundance of certain methanotrophs was a cause of macrofauna bioturbation affecting niches of methanotrophs inhabiting specific vertical layers of the oxygen-methane transition zone as seen in lake water [45], such as some methanotrophs potentially favoring submicromolar O_2_ conditions [36]. The SEM model showed a relationship between the relative abundance of methanotrophs and *pmoABC* transcripts. Likely a higher abundance of methanotrophs was reflected in an increase in RNA transcripts which has been observed previously in the study area [46, 47].

### Loss of infauna retains CH_4_ in the sediment favoring methanotrophy

We confirm that bioturbation by meiofauna and macrofauna increases oxygen penetration depth in the sediment [11, 12, 23, 48], which increases bacterial OM mineralization [20, 49] and allows for proliferation of aerobic bacteria as oxygen becomes available in sediment layers previously dominated by anaerobic processes [10]. However, our SEM analyses indicate that macrofaunal enhanced CH_4_ sediment–water flux had an effect on aerobic methanotrophs by limiting the availability of CH_4_ in the pore-water that these bacteria can grow on. Accordingly, aerobic methanotrophs have been found to be less abundant in coastal sediment in which pore-water CH_4_ concentrations are low [46]. The CH_4_ oxidation rate results further confirmed that sediment with low abundance of fauna increased the retention of CH_4_ in the sediment, which favored methanotrophic activity. Methane oxidation rates have previously been measured using radiotracers in shallow marine waters and sediments with lower CH_4_ concentrations leading to lower oxidation rates (see e.g. [7, 50]), however as far as we know this is the first study coupling such measurements to the presence of meio- and macrofauna. Considering a strong increase in CH_4_ oxidation was found in LM and not in the other treatments (after ^14^CH_4_ injection and 24 h incubation) it is unlikely that differences in CH_4_ pore-water and CH_4_ fluxes throughout the experiment are explained by methanogenic activity in deeper anoxic sediment. Methanogenesis has also been observed to co-occur with CH_4_ oxidation in a cryptic CH_4_ cycle in the sediment surface [51], and anaerobic methane oxidation by archaea can also be conducted via so-called reverse-methanogenesis [52]. However, in our study *mcrA* transcripts were close to zero with no differences between the treatments. Similarly to *mcrA* transcripts, Alpha-D-ribose 1-methylphosphonate 5-phosphate C-P lyase (*phnJ*), which is an essential protein utilized in oxic methane production via methylphosphonate (MPn) decomposition to methane [53], was also close to zero in the sediment surface. The SEM indicated that high abundances of meiofauna somewhat counterbalanced the higher CH_4_ fluxes seen in the presence of macrofauna. Previous studies have shown that meiofauna bioturbation changes redox profiles in the sediment [19], moving sulfate reduction pathways deeper into the sediment [22]. Such changes have the potential to affect anaerobic CH_4_ oxidation [50], CH_4_ production and diffusion rates of CH_4_ to the upper sediment, which was supported by the CH_4_ pore-water profiles, but such effects would need to be confirmed in future work. Finally, considering the similar O_2_ and CH_4_ sediment profiles between the CTRL and the HM, LMM, and HMM treatments, any drastic changes in redox condition because of our previously used [21, 23, 54] experimental design was unlikely.

Bivalve holobionts such as *M. balthica* have previously been shown *in vitro* to produce CH_4_ likely due to methanogenic archaea in their anoxic guts [24, 40]. Upscaling of previously reported bivalve CH_4_ production per biomass (direct bivalve production × sediment area) (see e.g. [24, 40]), indicate that bivalve production can only have constituted up to 14% of the areal benthic m^−2^ d^−1^ flux in the LMM and HMM treatments, suggesting that the largest part of the measured CH_4_ in our study was produced by methanogenic archaea living in the sediment. We also found that sediment with low meiofauna abundance and no macrofauna (LM treatment) increased the retention of CH_4_ in the sediment down to 4 cm. This was not detected in the other treatments, including HM that had no addition of macrofauna, but instead had an active flux of CH_4_ escaping the sediment. Potentially, this is related to changes in oxygen penetration depth and porosity due to meiofauna (and possibly grazing on bacteria) [14, 19, 23]. A previous study using a similar experimental setup found that meiofauna increased bacterial denitrification in coastal sediments [23], and here we show that these microscopic animals are also important for CH_4_ turnover. Aerobic methanotrophs have previously been shown to be more abundant in CH_4_-rich offshore coastal sediments compared with shallow inshore areas [46]. Higher biomass of benthic fauna in shallow marine waters [55] partly explains these findings by decreasing retention of pore-water CH_4_, which, in turn, suppresses the growth of aerobic methanotrophs.

### Outlook and implications

Our results indicate that a large part of CH_4_ emissions from shallow coastal waters are related to animal activity and complex ecological interactions between bioturbating infauna and benthic microbial community involved in CH_4_ turnover in the sediments. We provide further insight on the drivers of spatial and temporal variability of CH_4_ emissions in coastal areas [5, 6, 56, 57]. Our results suggest an important enhancement of the transport of CH_4_ from the sediment to the water column which also limits aerobic methanotrophs. Aerobic methanotrophs in oxic water have been described to oxidize up to 50% of the CH_4_ produced in marine shallow areas [7], and in our study we did observe methanotrophs and active CH_4_ oxidation in the oxic sediment surface of all treatments. This indicated active CH_4_ production in deeper anoxic sediment, however most of the produced CH_4_ had likely been oxidized by ANME before reaching the oxygenated sediment surface [8, 9]. Nevertheless, we measured an active CH_4_ flux from the sediment to the water, and especially sediments with low fauna had higher CH_4_ oxidation rates that decreased the CH_4_ sediment–water flux. Climate change and ongoing anthropogenic degradation of coastal ecosystems affects benthic fauna negatively [15], and our findings suggest that this will influence the dynamics of the CH_4_ turnover. Moreover, our results show that complex interactions between different taxonomic groups (macrofauna–meiofauna–bacteria) influence emission rates. Based on our results we suggest that biodiversity elements such as species characteristics and their abundance and biomass need further examination to fully understand CH_4_ emissions in coastal ecosystems.

## Materials and methods

### Sediment sampling

A total of 63 acrylic sediment cores (inner diameter 4.6 cm, length 30 cm) were collected 23 May 2022 on board R/V Augusta in the Storfjärden bay area (Finland). Cores with intact sediment and bottom water were by subsampled from a box corer (surface area: 1000 cm^2^, model 80.100–50, KC Denmark) at a site with 34 m water depth (Lat 59.8559, Long: 23.26695) previously shown to have high methanotrophic activity (station id: 10) [46]. Each collected core was closed with rubber stoppers and contained approximately half sediment (~14 cm) and half overlying bottom water (~14 cm). *In situ* bottom water temperature (6.5°C), dissolved O_2_ (11.9 mg L^−1^), and salinity (6.4 PSU) were measured with a ProODO probe (YSI, USA) and CTD CastAway CTD (SonTek, USA). Fifteen collected cores had pre-drilled holes (4 mm diameter, 1 cm vertical resolution) which were covered with silicone and water-resistant tape for later injection of ^14^CH_4_ tracer to measure CH_4_ oxidation rates. For three cores, the top 1 cm sediment layer (ca 17 mL sediment) was sliced on board the ship to obtain field data. The sediment was sliced into a flat 215 mL polypropylene container (207.0215PP, Noax Laboratory) and a 2 mL subsample was transferred using a cut-off 3-mL syringe (Henke-Ject) into a 20 mL gas tight glass vial (containing 4 mL 1 M NaOH) with butyl septa for pore-water CH_4_ analysis. The remaining sediment was homogenized and 2–3 mL of sediment was transferred into a 15 mL centrifuge tube that was flash frozen in liquid nitrogen, stored at −80°C and later used for RNA extraction. In addition, 1 mL sediment was transferred to aluminum trays for porosity and OM % estimation via drying and loss on ignition (LOI) analysis. The remaining sediment in the container was transferred to a 50 mL centrifuge tube that was stored at −20°C and later used to estimate meiofauna abundance. All remaining cores were stored at *in situ* bottom water temperature inside a climate room and were used the next day for the experimental setup. Finally, a Van Veen grab (20 × 20 cm) was used to collect additional sediment, which was sieved through a 1000 μM mesh. Individuals of the bivalve *M. balthica* were collected and kept in aerated bottom water until being added into sediment cores (see more details below).

### Meiofauna extractions and experimental setup

Meiofauna was extracted from the sediment the day after sampling as described in Bonaglia et al. [23], Näslund et al. [54]. In brief, the top 4 cm was sliced of each core, the slice was sequentially sieved through a 1000 and 40 μM sieve, and finally the animals were anesthetized by submersing the sediment in a 740 mM MgCl_2_ solution for 5 min [58]. The meiofauna was then separated from the sediment using a density extraction method as described in Broman et al. [30]. In brief, a Levasil silica gel colloidal dispersion solution (H.C. Starck) with a density of 1.21 kg m^−3^ was used to separate the animals by letting sediment particles settle to the bottom of an Erlenmeyer flask for 5 min. The retained animals in the top layer of the flask were collected on a 40 μM sieve and the procedure was repeated two more times. After the third isolation, the sieve was rinsed with sea water and the meiofauna material collected from two cores was combined and added into one of the cores (denoted HM), while the second core had no meiofauna material returned and was denoted as LM. Finally, finer sediment particles that had passed through the 40 μM sieve were returned to each core. This yielded treatments with a meiofauna abundance of: LM 46 ± 16, HM 173 ± 65, LMM 42 ± 8, HMM 214 ± 111, and CTRL 261 ± 100 (values show number of meiofauna on average ± SD 10^−3^ m^−2^ sediment per treatment).

Two individuals of *M. balthica* (body size 10–12 mm) were added to half of the manipulated cores to create treatments with HMM and LMM similarly to Bonaglia et al. [23]. As some cores had animals below the 4 cm layer previously sliced off during meiofauna extraction, the experimental setup yielded treatments with a macrofauna abundance of: LM 0 ± 0, HM 0 ± 0, LMM 2 ± 0, HMM 2 ± 1, and CTRL 1 ± 1 (values show number of animals on average ± SD in the cores per treatment, sediment core surface area: 16.6 cm^−2^). The macrofauna treatments were thus added with 1200 individuals m^−2^ sediment which is in the range previously reported for the Baltic Sea [38] and the Tvärminne Storfjärden bay [37]. *Macoma balthica* is typically found in the upper 0–5 cm sediment layer [59], and is one of the most common macrofaunal groups in the Baltic Sea and is known to be important in ecosystem functioning by e.g. enhancing the benthic-pelagic coupling, increase benthic respiration, and transport fresh OM into the sediment via bioturbation [39]. This resulted in an experimental design of LM, HM, LMM, and HMM (each treatment *n* = 12) as well as 12 unmanipulated control cores denoted CTRL. To keep the animals alive and active throughout the experiment, 3 mL of a solution containing 0.51 g dry Tetra Goldfish (Tetra) in 210 mL deionized water was added, and settled on the sediment surface, to each manipulated core (representing ca 4.4 g dw C m^−2^ sediment) [60]. Tetra Goldfish is a commercial fish food similar to Tetraphyll (Tetra) that has been previously shown to be a high-quality food source for benthic macrofauna [60].

The cores were then fully submersed in an aerated water bath (~95 L) consisting of *in situ* collected bottom water inside two incubation chambers. The cores were randomly distributed among the two chambers, and the chambers were placed inside a climate room set to an *in situ* bottom water temperature of ~6°C. Free rotating Teflon-coated magnets were attached in the water phase of each core and stirred externally using a rotating magnet. The cores were then acclimated in darkness for 10 d. Throughout the acclimation phase oxygen, temperature, and salinity were measured daily inside the two incubation chambers and had an average temperature of 5.9 ± 0.5°C, 13.2 ± 0.1 mg L^−1^ O_2_, and 5.4 ± 0.1 salinity (values show mean ± SD).

### Sediment slicing, porosity and oxygen sediment profiles

After 10 d of acclimation, the top 1 cm sediment layer was sliced for one core per treatment as described above for the field samples, with the exception that 5 mL was transferred to aluminum trays for porosity estimation. The cores were then sliced every cm down to 4 cm and sediment was collected for pore-water CH_4_ and porosity determinations as described above. Finally, sediment sampling for porosity determination was continued every 1 cm until the bottom of the core. Oxygen concentration profiles were measured using Clark-type microelectrodes (OX-50, Unisense) in three random cores per treatment with three microprofiles per core. Oxygen profiles of each treatment were obtained from averaged concentration profiles.

### Flux incubations and termination of the experiment

A total of 34 sediment cores were capped (*n* = 7 per treatment, except HMM *n* = 6) and incubated for 6 h to determine the CH_4_ and DIC sediment–water flux, as well as the O_2_ flux (i.e. oxygen consumption). Water samples were taken before and after incubation by transferring water into 20 mL glass vials that were poisoned with 100 μL 7 M ZnCl_2_ for CH_4_ samples, and filtering water through a 0.45 μM glass fiber filter into 12 mL Exetainer glass vials (Labco Scientific) for DIC measurement. All vials were filled, stored cold and upside down in darkness until analysis. Oxygen consumption in each core was measured by inserting microelectrode (OX-50, Unisense) into the water column before and after the incubations.

The day after the flux incubations, the experiment was terminated by slicing the top 1 cm sediment layer in each core, except for four cores per treatment (that had pre-drilled holes) which were kept for the ^14^CH_4_ radio tracer incubation (described in the section below). Sediment was collected as described above for the field samples. In addition, macrofauna in the sediment surface was collected, and the sediment left inside the core (i.e. below the top 1 cm) was sieved through a 1000 μM sieve to collect macrofauna potentially residing in deeper layers. Collected macrofauna was used to estimate abundance and biomass with more details described below (*n* = 7 cores per treatment).

### ^14^CH_4_ tracer incubation

A total of 20 cores (with 15 having pre-drilled holes) were used to estimate CH_4_ oxidation rates. Three cores per treatment were injected with 25 μL of a tracer solution of ^14^CH_4_ (activity per injection estimated at 65 Bq) prepared by dissolution of gaseous tracer in oxygen-free artificial seawater) in both the 1 and 2 cm sediment surface layers. Injection was conducted with a gas tight glass syringe using the side holes on the cores as described in Myllykangas et al. [50]. The remaining fourth core for each treatment was used as a control and was not injected with tracer. The cores were kept open at the top and submersed in the aerated bath. After 24 h, the 0–2 cm sediment surface of each core was sliced and funneled into 100 mL pre-weighed bottles containing 40 mL 4% NaOH (pH > 12, causing all DIC to be converted to CO_3_^2−^) [50]. The bottles were closed with a septum (PFTA, 5 mm) and gas tight lid cap, re-weighed with wet sediment inside to know the exact sediment volume sliced, stored upside down, and kept dark in room temperature until analysis. The controls were sliced in a similar manner and ^14^CH_4_ tracer was added directly into the bottles after slicing to estimate the amount of tracer added and potentially lost during storage (<1 week).

The samples were analyzed following the protocol of Myllykangas et al. [50]. In brief, residual ^14^CH_4_ tracer in the headspace of the bottles not converted to ^14^CO_2_ during the incubation was captured through combustion in a tube furnace inside a glass tube containing copper oxide (850°C, Nabertherm). The combusted CO_2_ was trapped using CO_2_-absorbant (2-phenylethylamine and 2-methoxyethanol, 1:7 v/v) and analyzed using a liquid scintillation counter (Wallac 1415) after addition of Ultima Gold scintillation cocktail (Perkin Elmer). The sediment in the bottles were then used to measure the amount of injected ^14^CH_4_ oxidized to ^14^CO_2_ by adding 6 M HCl (pH < 1) that caused DIC to be converted to CO_2_ and trapped using CO_2_-absorbant (0.5 M NaOH, 2-phenylethylamine, 1:1 v/v). The samples were then analyzed using liquid scintillation. The potential CH_4_ oxidation rate was calculated according to Treude et al. [61] following the modifications by Myllykangas et al. [50] based on the amount of ^14^CO_2_ that was produced in relation to the total ^14^C pool (Equation 1):


$$ Oxidation\ rate=\frac{{}^{14}C{O}_2\times{CH}_4}{\Big({}^{14}C{O}_2+{}^{14}C{H}_4\Big)\times t\times v} $$

where CH_4_ is the total concentration of nmol CH_4_ measured at time-zero, ^14^CO_2_ and ^14^CH_4_ are the activities (disintegrations per minute, DPM) measured after incubation, and *t* and *v* are incubation time (days) and sediment volume (cm^3^), respectively. Average DPM values measured in the blanks were subtracted from the ^14^CO_2_ values. A sample was considered active if values were within × 3 SD of the blanks. The ^14^CH_4_ values were corrected for any potential gas loss during storage by using the ratio between time-zero and post-incubation CH_4_ concentrations. Tracer recoveries were generally >90%. CH_4_ turnover was calculated by estimating the proportion of injected ^14^CH_4_ tracer retrieved from the sum of ^14^CO_2_ and ^14^CH_4_ (Equation 2):


$$ Turnover=\frac{{}^{14}C{O}_2}{\left({}^{14}C{H}_4+{}^{14}C{O}_2\right)}$$

### Chemical analyses

DIC water samples were analyzed using a DIC analyzer (Apollo SciTech, model AS-C5). The instrument was calibrated using repeated measurements of certified reference material from the Scripps Institution of Oceanography (CRM batch number 191). One LM vial for DIC measurement broke during the transportation and is therefore missing in the data. CH_4_ water and sediment pore-water samples were quantified by headspace analysis using a gas chromatograph (GC Trace 1300, Thermo) equipped with an autosampler (TriPlus RSH, Thermo), a non-polar PLOT column (TracePLOT TG-BOND Q, Thermo), and a flame ionization detector. Certified standards of 1.86and 49.82 ppm CH_4_ (Air Liquide Gas) were injected and used for calibration. The ppm concentrations were converted into molar concentrations, adjusting for sediment porosity, using the ideal gas law. Sediment porosity was determined by weighing wet and dry sediment (48 h at 70°C). This was followed by LOI analysis by measuring the weigh difference before and after igniting the dry sediment at 550°C for 5 h.

### Infauna quantification

Macrofauna collected at the end of the experiment from all cores were identified, counted, and biomass estimated from complete animals via weight loss (dried at 60°C for 24 h). Frozen sediment taken from the 0–1 cm layer was thawed (2–8 mL) and used to isolate meiofauna using density extraction as described earlier. The isolated meiofauna was sorted, identified, and counted using a ×50 binocular stereomicroscope.

### RNA extraction and sequencing

RNA was extracted from the samples (~2 g sediment) using the RNeasy PowerSoil Total Kit (Qiagen) following the manufacturer’s instructions. DNA contamination was removed with the TURBO DNA-free kit (Invitrogen) and confirmed to be DNA free by gel electrophoresis. The extracted RNA was delivered to SciLifeLab, Stockholm where libraries were prepared with the TruSeq Stranded mRNA kit (Illumina) excluding the poly-A selection step (i.e. libraries contained total RNA). The libraries were sequenced on a NovaSeq 6000 (Illumina) S4 lane with a 2 × 150 bp setup and the output data were demultiplexed by the sequencing facility.

### Quantitative reverse transcription PCR

RNA samples were normalized to 10 ng μL^−1^, reverse transcribed into cDNA using the AccuScript High Fidelity 1st Strand cDNA Synthesis kit (Agilent) using the supplied random hexamer primers. The cDNA was then used as input material in RT-qPCR reactions containing primers 16S rRNA 515F and 805R [62, 63]. RT-qPCR reactions contained 1 μL of cDNA were used with 7 μL H_2_O, 10 μL SYBR GREEN (LightCycler 480 SYBR Green I Master kit, Roche), and 1 μL of each primer. The qPCR program consisted of an initial denaturation at 95°C for 5 min, followed by 45 cycles of denaturation 95°C 30 s, annealing 60°C 30 s, and elongation 72°C for 15 s. The ramp rate was set to 4.40°C/s except for the annealing step that was 2.20°C/s. Technical duplicates were performed for each sample and the average Ct value was used.

### Bioinformatics of the RNA-seq data

Total RNA sequencing yielded on average 71.4 million paired-end reads per sample (min: 54.5, max: 91.4). Quality trimming was conducted by: (i) removing Illumina adapters using SeqPrep 1.2 with default settings targeting the adapter sequences [64]; (ii) remove any leftover PhiX control sequences by mapping the reads to the PhiX genome (NCBI Reference Sequence: NC_001422.1) using bowtie2 2.3.5.1 [65], and (iii) remove low quality and short reads using Trimmomatic 0.39 with settings: LEADING:20, TRAILING:20, and MINLEN:80 [66]. The data were then used to produce quality reports with FastQC 0.11.9 [67] and combine the reports with MultiQC 1.12 [68]. The quality trimmed data had on average 69.5 million paired-end reads (min: 53.2, max: 89.1), an average read length of 146 bp (min: 145, max: 147), and each sample an average read Phred score of 36 (min: 36, max: 36).

Taxonomic classification was conducted by extracting small subunit rRNA (16S and 18S SSU rRNA) reads from the quality trimmed data using SortMeRNA 4.3.6 [69] by using the SSU reference sequences in the supplied database (smr_v4.3_default_db.fasta). The SSU reads were annotated using Kraken 2.0.9 [70] using default settings against the SILVA database (download date: 1 September 2022) with a paired-end setup (setting: --paired). This was followed by using Bracken 2.7 [71] to distribute reads to genus level and be able to compare relative abundances (settings: -r 150 -l G -t 10, i.e. read length of 150 bp, genus level, and a threshold of 10 counts per genus). The Bracken reports were combined and converted to a tab delimited text file with the python packages kraken-biom 1.0.1 [72] and biom 2.1.7 [73] as described earlier [74]. Prokaryotic (i.e. 16S rRNA classifications) were extracted from the dataset and on average there were 26.0 million counts per sample (min: 19.3, max: 34.0). The data were then analyzed in the software Explicet 2.10.5 [75] and normalized as relative abundances (%).

The functional annotation followed a similar approach as outlined in the SAMSA2 pipeline [76]. The quality trimmed reads were merged using PEAR 0.9.10 with default settings [77] which yielded on average 49.9 million merged reads per sample (min: 37.1, max: 62.1) with an average read length of 207 bp (min: 200, max: 213). Forward reads not merged were concatenated with the merged reads as recommended in the SAMSA2 pipeline [76]. This was followed by extracting non-rRNA sequences from the data using SortMeRNA and the default supplied database (smr_v4.3_default_db.fasta) (~2% of the data, i.e., ~1.3 million reads per sample). The non-rRNA reads were annotated using DIAMOND 2.0.14 [78] against the NCBI NR database (download date: 3 January 2023) with an e-value threshold of 1e^−10^. The output NCBI accessions were linked to KEGG KO classifications using default settings with the daa-meganizer tool and MEGAN database (MEGAN database: megan-map-Feb2022) supplied with MEGAN 6 Ultimate Edition 6.24.5 [79, 80]. Here we used default settings with the meganizing tool, which excludes blast hits with a bit score below 50, and hits outside the top 10% of the highest bit score. Eukaryota and virus classifications were excluded from the dataset by supplying a contamination file with the daa-meganizer tool (setting: -cf). The meganized .daa files were combined into a .megan file containing raw counts using the MEGAN supplied tool compute-comparison. The data were imported into MEGAN and all assigned KEGG KOs were extracted, and counts normalized as counts per million mapped reads (CPM, i.e. relative proportion × 1 million). Finally, to estimate the % of pmo in pmo/amo KEGG KO classifications, the MEGAN supplied tool read-extractor was ukksed to extract reads classified as *pmoABC*/*amoABC* in the KEGG database (KEGG KOs: 10944, 10945, 10946). These reads were annotated using BLASTX (Blast version 2.12.0+, e-value threshold = 1e^−10^) [81] against the UniProtKB-SwissProt database (download date: 21 December 2022). The number of hits per *pmoAB* or *amoAB* genes were then used to calculate the proportion of pmo (note that *pmoC* or *amoC* are not available in the curated UniProtKB-SwissProt database).

### Statistics

Shapiro–Wilk tests, using the function *shapiro.test* in R, was used to see if data followed a normal distribution, and Levene’s test were used to test the homogeneity of variance using the function *leveneTest* using the R package car 3.1.0 [82]. Differences between treatments were tested with one-way ANOVA and *post hoc* Tukey tests using the functions *aov* and *TukeyHSD* in R 4.2.0 [83]. Variables not following the primary assumptions of ANOVA were transformed, this included log transformation of meiofauna abundance, CH_4_ pore-water and CH_4_ oxidation rate, and square root transformation of CH_4_ flux and DIC flux. Macrofauna biomass, O_2_ penetration depth in the sediment, and O_2_ consumption in the water phase could not be transformed to a normal distribution and were therefore tested using non-parametric Dunn tests with Benjamini–Hochberg p-adjustment using the *dunn.test* function in the R package dunn.test 1.3.5 [84]. Spearman correlations between variables were tested and plotted using the function *corrplot* in the R package corrplot [85]. The RNA-seq taxonomy and transcripts data were analyzed using the Bray–Curtis dissimilarity index and visualized as NMDS using the software Past 4.11 [86]. PERMANOVA (9999 permutations) were conducted to test differences in beta diversity (Bray–Curtis) between the treatments, including pairwise tests between treatments with Bonferroni corrected *P* values, using the software Past. To test which methanotrophic genera contributed the most to the dissimilarity in Bray–Curtis beta diversity SIMPER tests were conducted using the function *simper* with the R package vegan 2.6.2 [87].

A piecewise SEM approach was used to examine the direct and indirect effects of biotic and abiotic variables measured in our experiments on CH_4_ fluxes. More specifically, we were interested in quantifying both the direct effect of macrofauna and meiofauna on CH_4_ fluxes and indirect effects on named fluxes through its impact on the abundance and activity of aerobic methanotrophs. The SEM model was constructed combining linear models with the function *psem* in the R package piecewiseSEM 2.3.0 [88]. Models with other distributions (e.g. gamma and poisson) were also tested but yielded similar Akaike’s information criterion (AIC) values. The full model with expected paths (including data on macrofauna biomass, meiofauna abundance, CH_4_ flux, CH_4_ pore-water, O_2_ flux, methanotrophs relative abundance, and *pmoABC* transcripts) based on the literature was tested. Samples that contained data from all variables could be used in the model (*n* = 7 for each variable per treatment, i.e. *n* = 35 per variable included in the model). O_2_ flux was used to substitute oxygen penetration depth data due to higher number of replicates per treatment (*n* = 7 vs *n* = 3). CH_4_ oxidation rates were measured on separate cores injected with ^14^CH_4_ (*n* = 3 per treatment) and could therefore not be included in the model. In addition, a most parsimonious SEM model with the lowest AIC was built by sequentially removing non-significant paths from the full model, until all remaining paths were significant and no remaining “missing” paths were detected. Overall model fit was tested using Shipley’s d-separation test via a Fisher’s *C* statistic and *χ*^2^-based *P* value [89, 90].

## Supplementary Material

Supplementary_Information_wrae013

Supplementary_Data_1_wrae013

Supplementary_Data_2_wrae013

Supplementary_Data_3_wrae013

Supplementary_Data_4_wrae013

Supplementary_Data_5_wrae013

## Data Availability

The data that support these findings are available in the manuscript and supplemental files. The supplemental files include Supplementary Data 1 (abiotic data, macrofauna biomass, and RT-qPCR results), Supplementary Data 2 (RNA-seq taxonomy results), Supplementary Data 3 (RNA-seq transcript annotation results), Supplementary Data 4 (bioinformatics statistics such as number of reads before and after quality trimming), and Supplementary Data 5 (results of statistical tests and SEM models). The raw sequencing data have been uploaded to the NCBI BioProject PRJNA917468.
