# Supplementary material for: Biotic interactions between benthic infauna and aerobic methanotrophs mediate methane fluxes from coastal sediments
Source: ISME J. 2024 Jan 31;18(1):wrae013. doi: 10.1093/ismejo/wrae013 (PMC10942774; doi:10.1093/ismejo/wrae013)
Supplement: Supplementary_Information_wrae013 [file supplementary_information_wrae013.docx]

**Biotic interactions between benthic infauna and aerobic methanotrophs mediate methane fluxes from coastal sediments**

Elias Broman, Markus Olsson, Adele Maciute, Daniel Donald, Christoph Humborg, Alf Norkko, Tom Jilbert, Stefano Bonaglia, Francisco J.A. Nascimento

**Supplementary Information**

*Captions to supplementary data files*

**Supplementary Data 1** The various sheets in the spreadsheet file shows: 1) data for CH_4_ and DIC fluxes and O_2_ consumption, CH_4_ pore-water, RT-qPCR data, and OM %; 2) data from the ^14^CH_4_ tracer experiment; 3) CH_4_ pore-water depth profiles; 4) Oxygen concentration depth profiles; 5) Macrofauna abundance and biomass; and 6) Meiofauna abundance.

**Supplementary Data 2** The file shows the assigned SILVA taxonomy after the Kraken2 + Bracken2 analysis of the RNA-seq data (extracted 16S rRNA reads). The table shows raw sequence counts.

**Supplementary Data 3** KEGG classifications of the RNA transcripts annotated and assigned with the DIAMOND + MEGAN softwares. The table shows raw sequence counts.

**Supplementary Data 4** The file shows various statistics from the bioinformatic analysis of the RNA-seq data. For example, sequence facility sample ID, sample names, number of reads after sequencing, read length, number of reads before and after quality trimming, number of 16S rRNA reads taxonomically classified, etc.

**Supplementary Data 5** The various sheets in the spreadsheet file shows results form: 1) ANOVA with Tukey Posthoc tests; 2) Dunn tests macrofauna biomass; 3) Dunn tests of O_2_ penetration death and O_2_ consumption; 4) Dunn tests of 16S rRNA methanotrophic genera; 5) PERMANOVA tests between treatments based on the Bray-Curtis dissimilarity; and 6) results from the SIMPER analysis.

*Supplementary Figures*

**
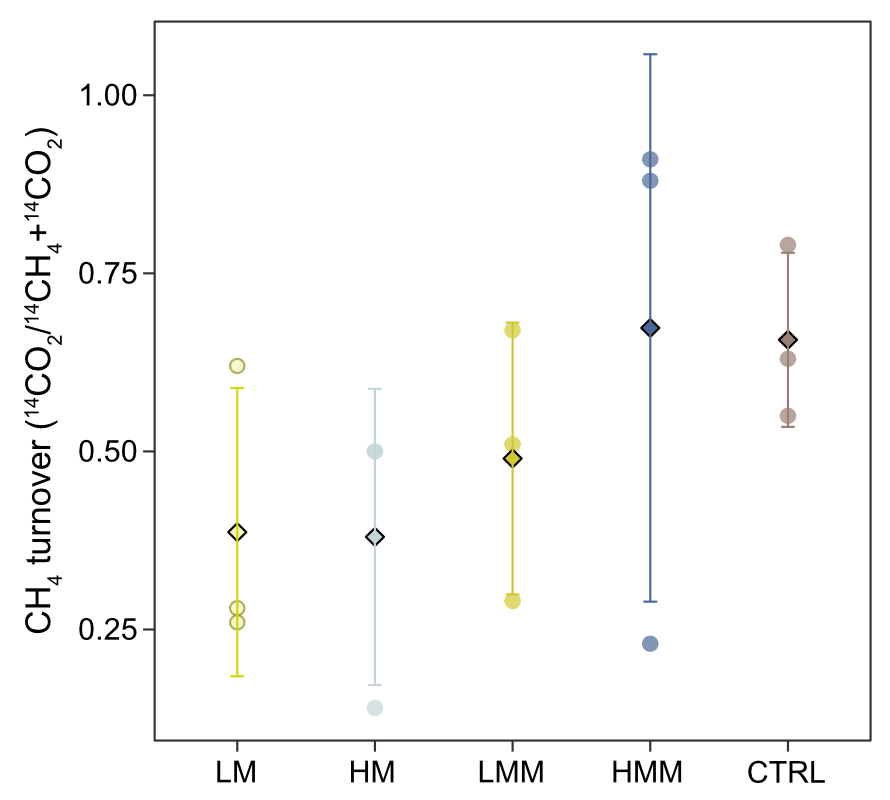
**

**Supplementary Figure 1** Calculated CH_4_ turnover based on measured ^14^CO_2_ and ^14^CH_4_ from the ^14^CH_4_ tracer incubation. ^14^CH_4_ was injected in the top 0–2 cm sediment layer (*n* = 3 per treatment). The dots denote individual incubated sediment cores, diamonds denote mean values, and the error bars show SD.


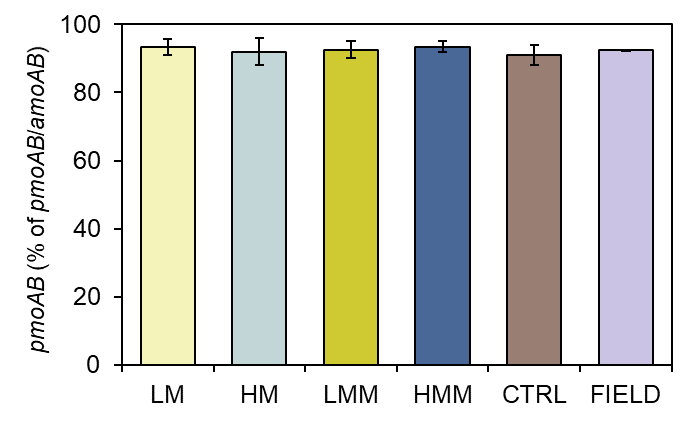


**Supplementary Figure 2** Proportion of *pmoAB*/*amoAB* KEGG classified reads classified as *pmoAB* against UniProtKB-SwissProt (*n* = 8 per treatment, except field *n* = 3). The information was used to estimate the % of reads beloning to particulate methane monoxygenase.


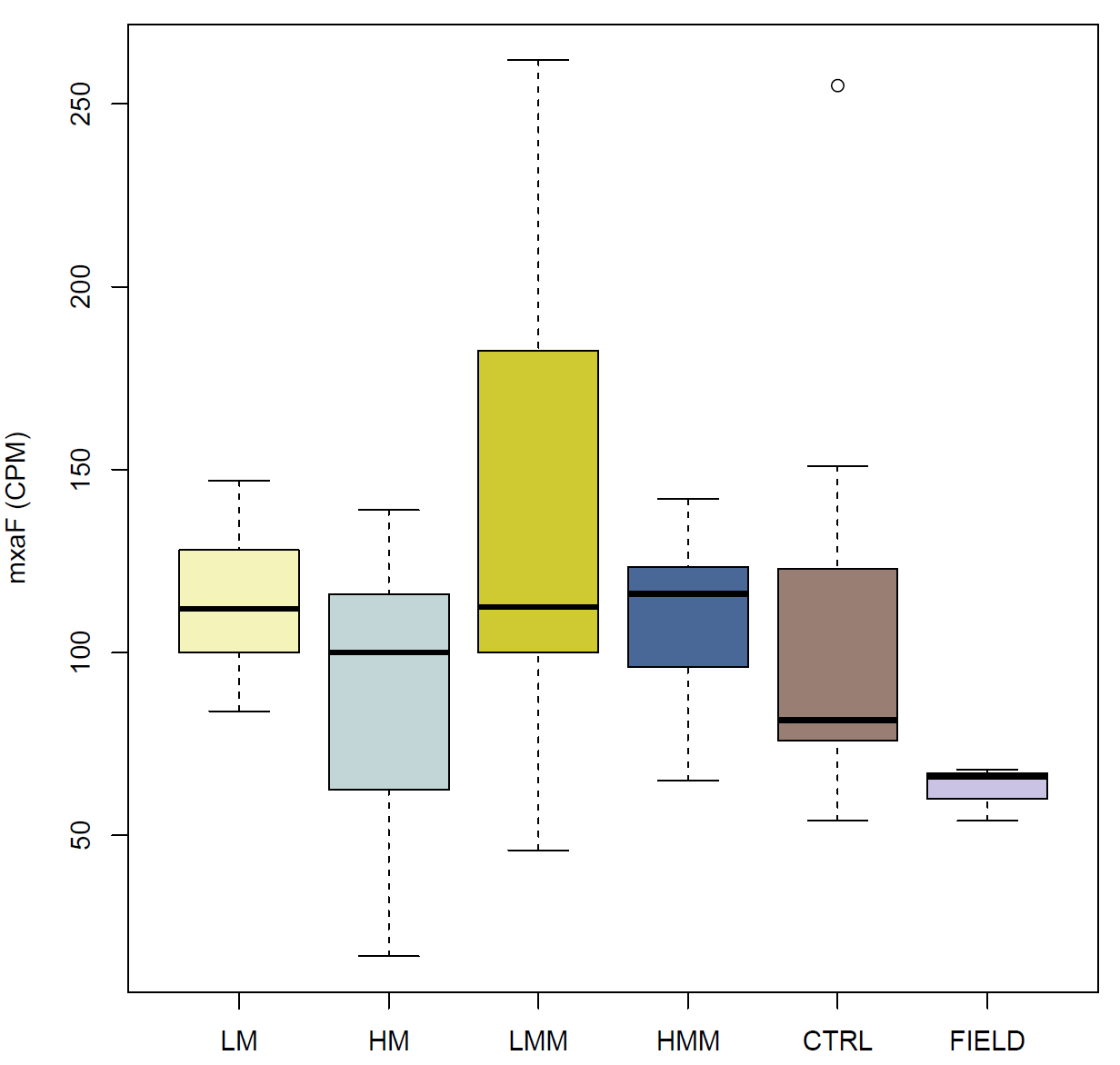

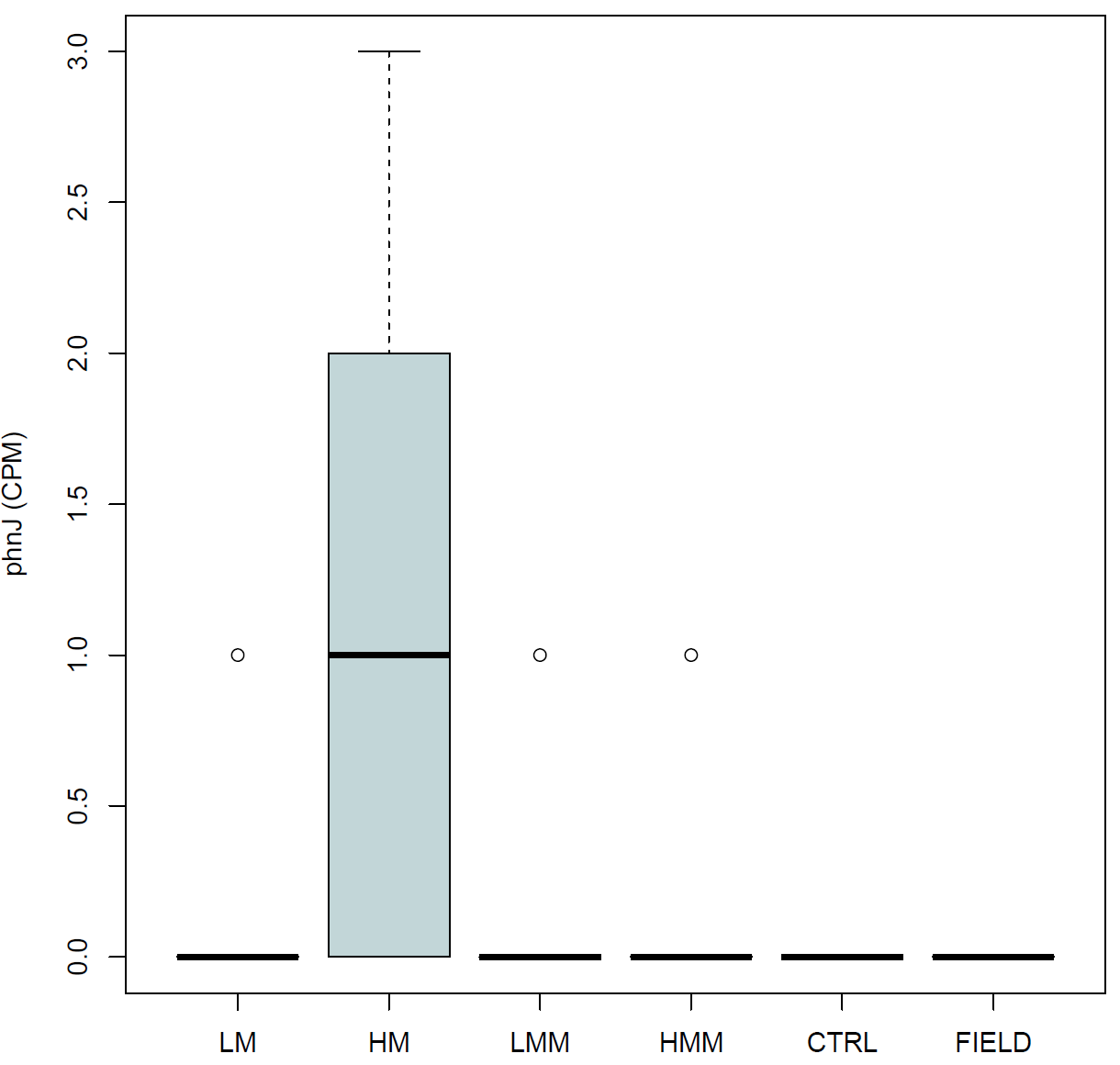

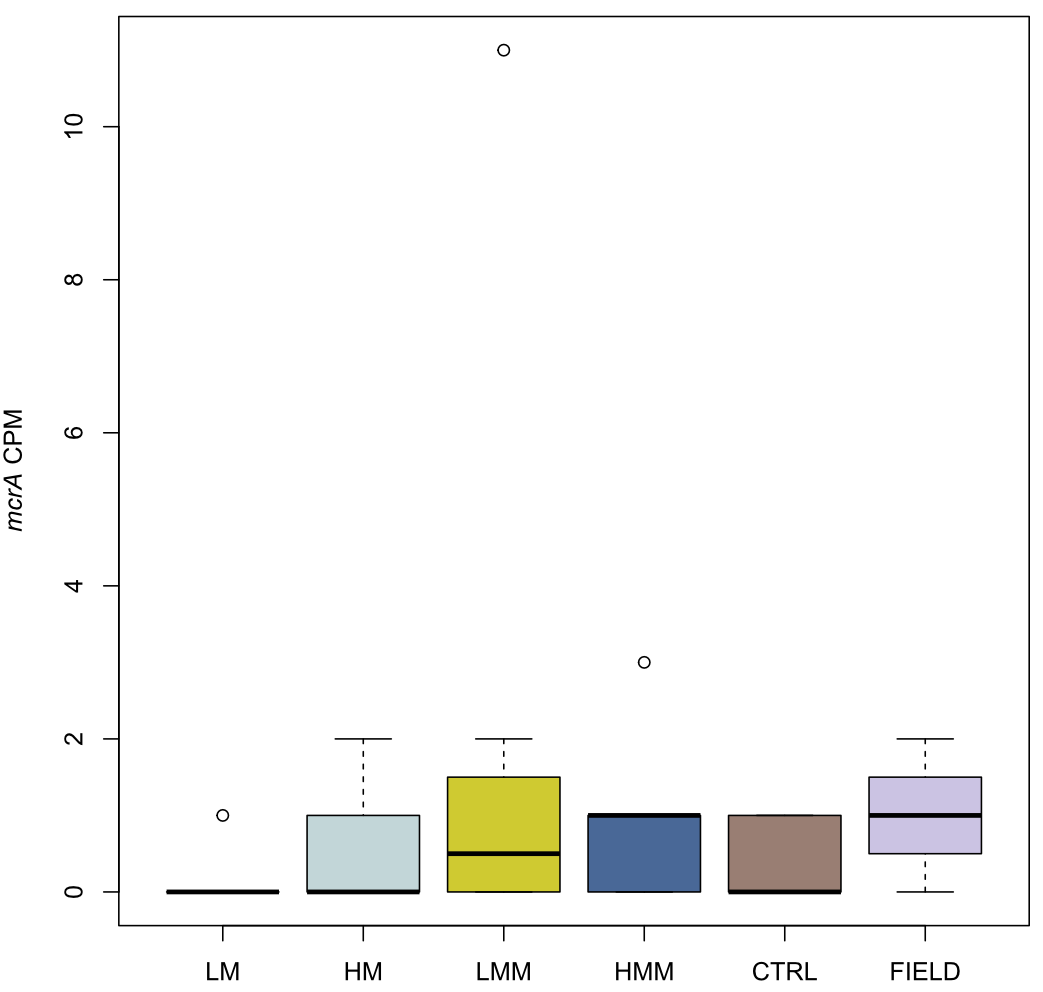


**Supplementary Figure 3** Methanol dehydrogenase (*mxaF*), Alpha-D-ribose 1-methylphosphonate 5-phosphate C-P lyase (*phnJ*), and Methyl-coenzyme M reductase alpha subunit (*mcrA*) transcripts abundance normalized as CPM-values.

**
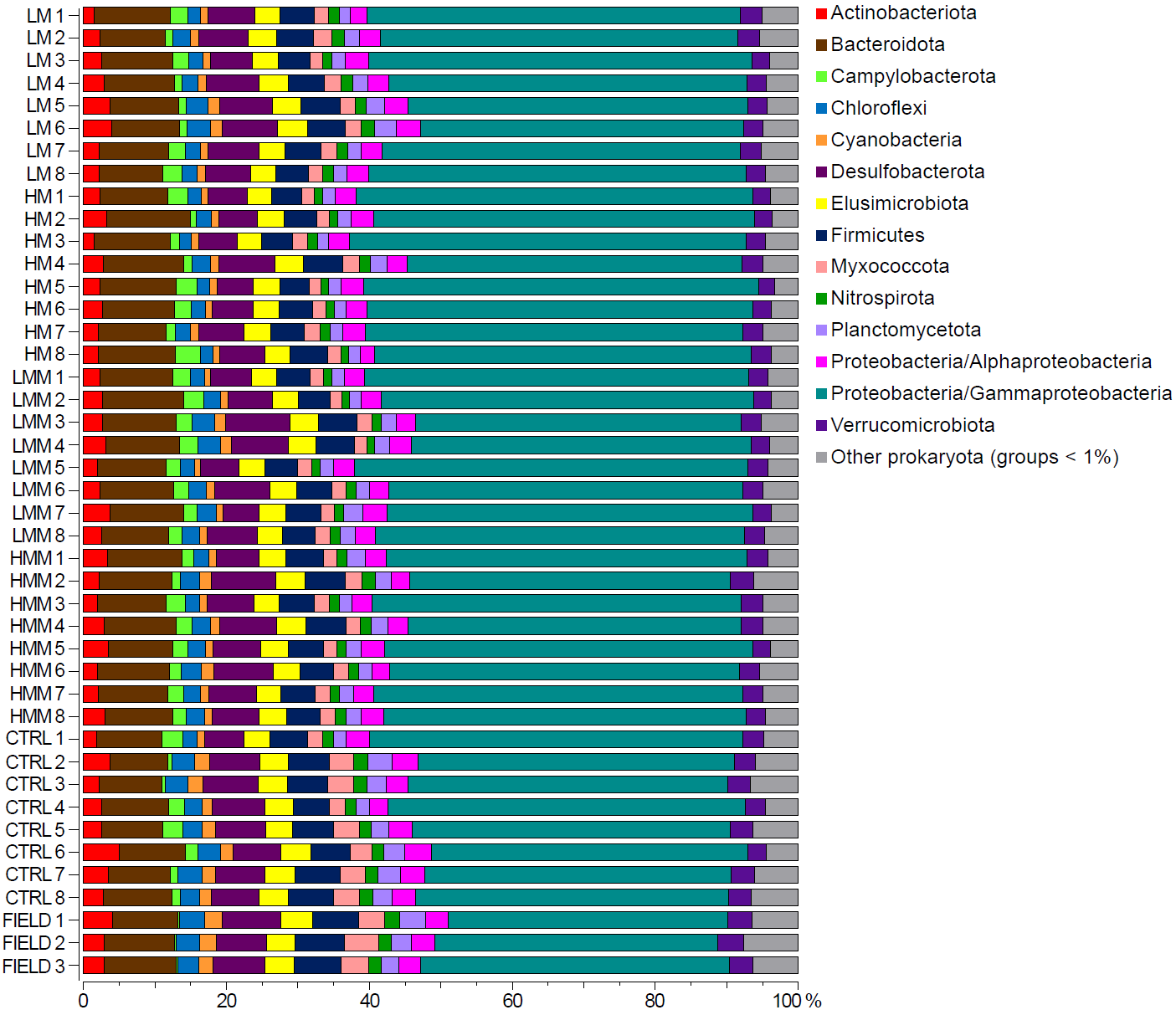
**

**Supplementary Figure 4** RNA-seq prokaryotic 16S rRNA taxonomy based on the software combo Kraken2+Bracken2 against the SILVA database. The figure shows phyla and Proteobacyteria divided into classes.

**
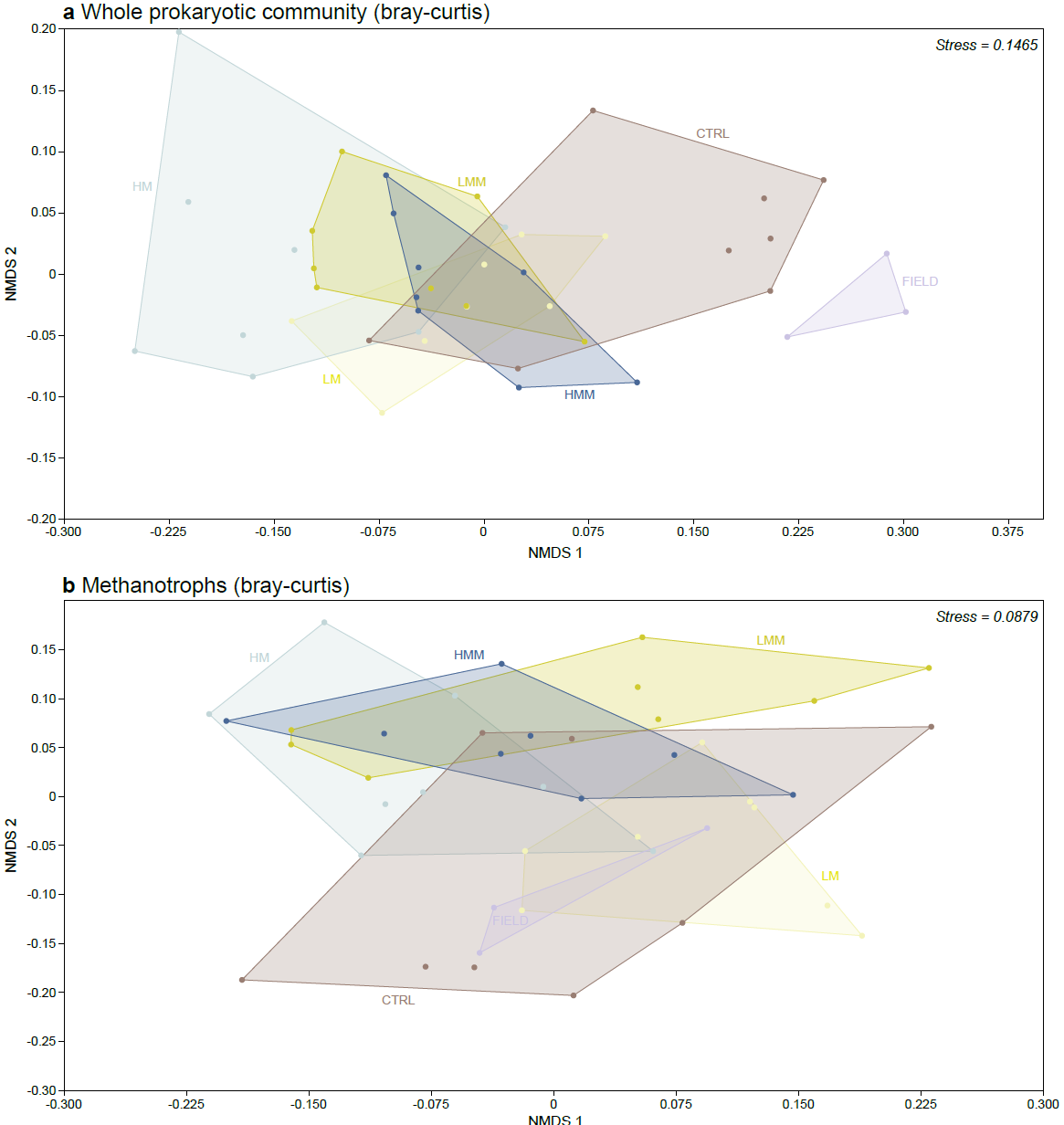
**

**Supplementary Figure 5** NMDS plots showing the difference in 16S rRNA beta diversity (Bray-Curtis) between the treatments for A) the whole prokaryotic community and B) methanotrophs.

**
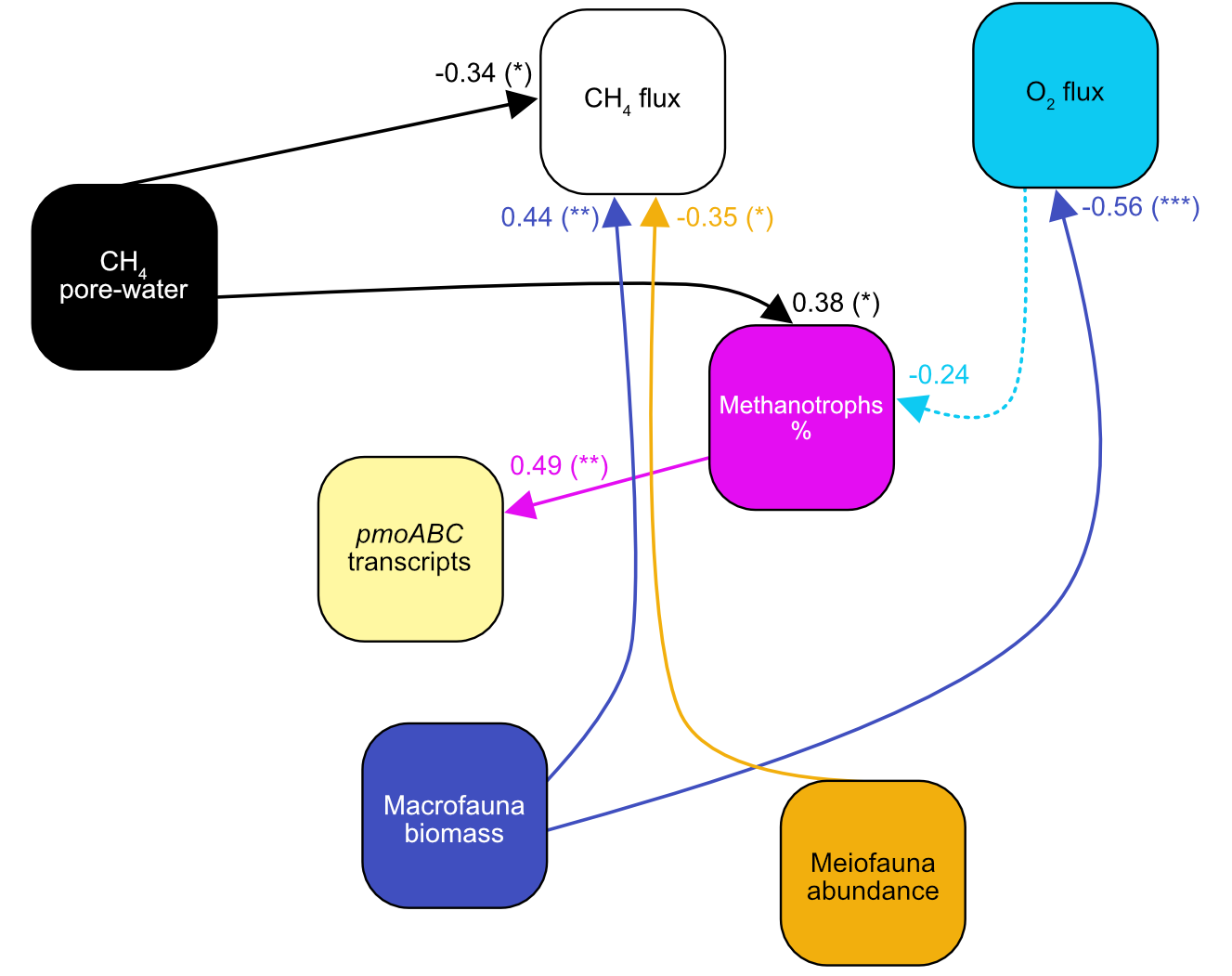
**

**Supplementary Figure 6** Parsimonous SEM model. Solid line arrows show significant effects. The numbers show standardized coefficients while the stars denote *P*-values < 0.05 (*), < 0.01 (**), and < 0.001 (***).

**
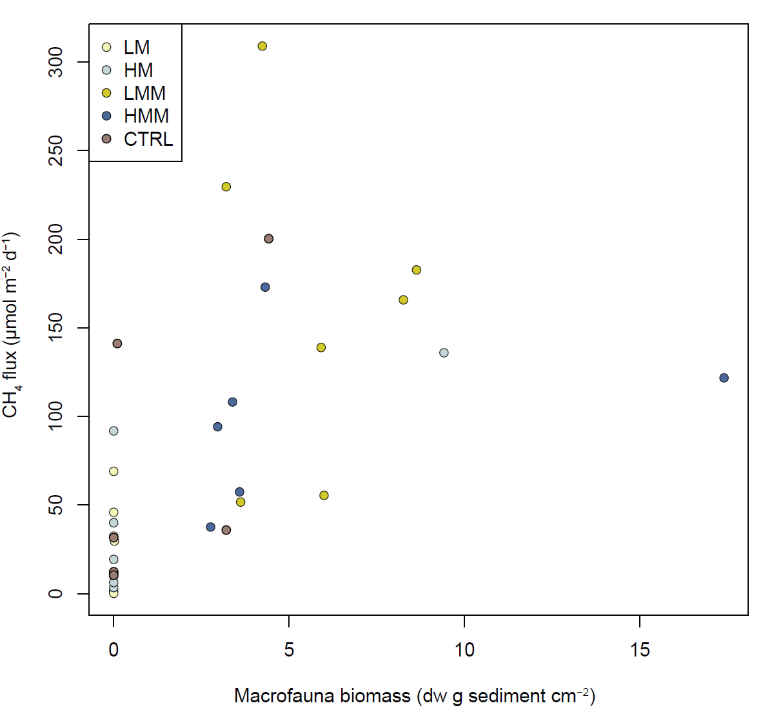

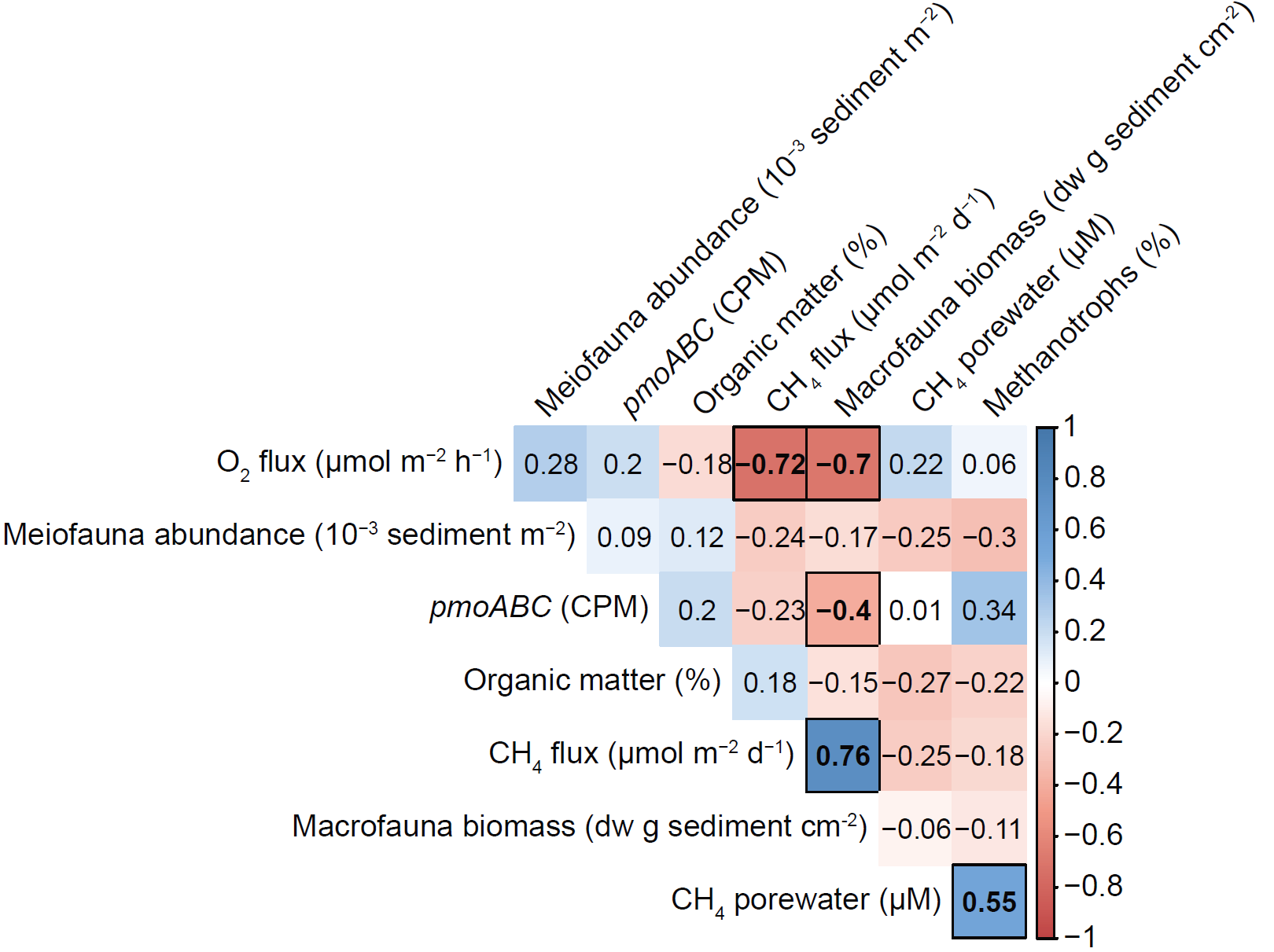
**

**
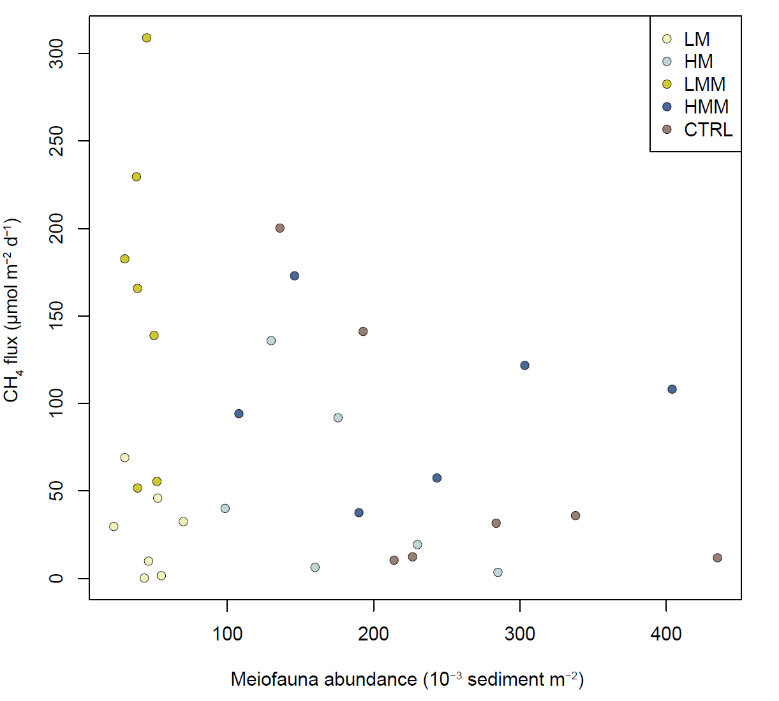
**

**Supplementary Figure 7** The top panel shows a correlogram of spearman correlations between measured variables. Bold text in boxes with a black outline indicate statistically significant correlations (*p* < 0.05). The values show spearman’s *rho*. The bottom panel shows scatter plots of CH_4_ flux (y-axis) and macrofauna biomass and meiofaunande abundance (x-axes).
